# Quantitative assessment of liver fibrosis by digital image analysis reveals correlation with qualitative clinical fibrosis staging in liver transplant patients

**DOI:** 10.1371/journal.pone.0239624

**Published:** 2020-09-28

**Authors:** Kun Jiang, Mohammad K. Mohammad, Wasim A. Dar, Jun Kong, Alton B. Farris

**Affiliations:** 1 Department of Pathology, Emory University, Atlanta, Georgia, United States of America; 2 Department of Pathology, University of South Florida, Tampa, Florida, United States of America; 3 Department of Surgery, The University of Texas Health Science Center, Houston, Texas, United States of America; 4 Department of Mathematics and Statistics, Georgia State University, Atlanta, Georgia, United States of America; 5 Department of Computer Science, Georgia State University, Atlanta, Georgia, United States of America; 6 Department of Computer Science, Emory University, Atlanta, Georgia, United States of America; 7 Department of Biomedical Informatics, Emory University, Atlanta, Georgia, United States of America; 8 Winship Cancer Institute, Emory University, Atlanta, Georgia, United States of America; University of Tsukuba, JAPAN

## Abstract

Technologies for digitizing tissues provide important quantitative data for liver histopathology investigation. We aimed to assess liver fibrosis degree with quantitative morphometric measurements of histopathological sections utilizing digital image analysis (DIA) and to further investigate if a correlation with histopathologic scoring (Scheuer staging) exists. A retrospective study of patients with at least two post-liver transplant biopsies having a Scheuer stage of ≤ 2 at baseline were gathered. Portal tract fibrotic percentage (%) and size (μm^2^) were measured by DIA, while clinical fibrosis score was measured by the Scheuer system. Correlations between DIA measurements and Scheuer scores were computed by Spearman correlation analysis. Differences between mean levels of fibrosis (score, size, and percentage) at baseline versus second visit were computed by Student’s t-test. *P* values < 0.05 were considered significant. Of 22 patients who met the study criteria, 54 biopsies were included for analysis. Average levels ±standard error [S.E.] of portal tract fibrotic percentage (%) and size (μm^2^) progressed from 46.5 ± 3.6% at baseline to 61.8 ± 3.8% at the second visit (P = 0.005 by Student’s t-test), and from 28,075 ± 3,232 μm^2^ at base line to 67,146 ± 10,639 μm^2^ at the second visit (P = 0.002 by Student’s t-test), respectively. Average levels of Scheuer fibrosis scores progressed from 0.55±0.19 at baseline to 1.14±0.26 at the second visit (P = 0.02 by Student’s t-test). Portal tract fibrotic percentage (%) and portal tract fibrotic size were directly correlated with clinical Scheuer fibrosis stage, with Spearman correlation coefficient and P value computed as r = 0.70, P < 0.0001 and r = 0.41, P = 0.002, respectively. Digital quantitative assessment of portal triad size and fibrosis percentage demonstrates a strong correlation with visually assessed histologic stage of liver fibrosis and complements the standard assessment for allograft monitoring, suggesting the utility of future WSI analysis.

## Introduction

Detecting post-transplant liver fibrosis at an early stage with a single investigation remains a clinical and pathological challenge because liver diseases are usually silent except in the extremes of presentation, such as in acute hepatitis or in cirrhosis (stage 4 fibrosis) [1]. Liver fibrosis is a predictor of liver disease progression and mortality, and current guidelines recommend screening for complications of cirrhosis once pathologists detect stage 3 fibrosis [2]. There is still no Food and Drug Administration (FDA)-approved treatment for advanced liver fibrosis, and none of the pharmacological agents on the market have shown noteworthy efficacy in reversing significant fibrosis [3]. Liver transplantation (LT) remains the only treatment option for patients with this devastating disease [3]. Thus, protecting the graft after liver transplant by early detection of lower stage fibrosis (stage 1–2) when intervention is feasible is critical to avoid losing the graft and eventually having a re-transplant. Among all etiologies of end-stage liver disease, nonalcoholic steatohepatitis (NASH)/nonalcoholic fatty liver disease (NAFLD) and hepatitis C virus (HCV) remain the leading causes of liver transplant [3, 4]. Moreover, recurrent NAFLD develops in 30–60% of patients following LT, and the recurrence rate of diseases (post-transplant) is up to 70% in cancer, and 60–90% in HCV [4]. The latest is likely to prevail as an important cause for liver transplantation in the foreseeable future despite the decrease in HCV-related end-stage liver disease due to the impact of antivirals for HCV and improved prevention and treatment for HCV-related HCC [5, 6]. Moreover, there is an increase in the incidence of nonalcoholic and alcoholic fatty liver disease becoming the most frequent causes of liver transplantation both for end-stage liver disease and hepatocellular carcinoma. Remarkably, during the past 10 years, the prevalence of NAFLD as an indication for LT has increased by 170% [3, 4]. Fibrosis regression in both HCV and NASH is possible in the early disease stages, while only minimal improvements can be achieved in the advanced stage. Thus, it is critical to prevent second injury and detect early stage fibrosis in postoperative monitoring of liver histology.

Liver biopsy is the standard method for liver histopathology assessment, providing valuable information of the fibrosis degree, parenchymal integrity, inflammation degree and pattern, bile duct status, and deposition of materials and minerals in the liver [7]. Post-transplant biopsies are performed to assess the necro-inflammatory activity (grading) and the severity of fibrosis (staging), evaluate the therapeutic response, and exclude other hepatopathy. Undoubtedly, the information obtained by liver biopsy is critical; however, there are concerns regarding the accuracy and safety of liver biopsy in the posttransplant settings [8]. Liver biopsies may not completely represent the stage of liver fibrosis because of sampling error and semi-quantitative measurement [9]. In clinical practice, pathologists frequently encounter severe problems of large inter- and intra-observer variation [10]. Interpreter errors account for 15–33% of variability in staging of fibrosis, and 10% of grading of necroinflammation [10]. Increasing the sample size of liver biopsy was a suggestion to achieve an ideal diagnostic accuracy, but it is clinically infeasible and sometimes dangerous to pursue [10].

Serum-based laboratory biomarkers and radiological investigations may help in the assessment of liver fibrosis; however, they have limitations in the assessment of liver fibrosis degree, particularly for early/intermediate stage cases and for the appreciation of cases with progression or regression. Magnetic resonance elastography (MRE) is also generally thought to be a reliable method for assessing liver fibrosis. Fibroscan (also known as “transient elastography”) is an ultrasound-based method for the assessment of liver fibrosis that generally has a lower sensitivity and specificity than MRE and is not as effective in the assessment of mild to moderate fibrosis. Despite the utility of these noninvasive techniques, particularly in the exclusion of cirrhosis, liver biopsies are still considered to be the “gold” standard for diagnosing fibrosis. Thus, clinical alternatives are not always considered to be proper surrogates to liver biopsy histopathologic evaluation [7, 11–15].

Currently, several histological staging systems for liver biopsy evaluation have been utilized in clinical practice. They can improve the diagnostic accuracy of liver fibrosis, even though liver biopsy is considered an invasive intervention [16]. However, all these histological scores and staging systems, such as Knodell, METAVIR, Ishak, and Scheuer systems, provide description of architectural changes and sites of fibrosis based on several categories (ranging from no fibrosis to cirrhosis) in a discrete, stepwise manner, without taking specifically into account quantitative fibrotic changes [17]. Meanwhile, these tools are subjective and associated with considerable intra-observer and inter-observer variability as well [18, 19]. Therefore, the development and usage of a novel histological index that quantitates fibrosis and relates to clinical outcome is required and would greatly improve the liver biopsy value.

Computer-assisted digital image analysis (DIA), a morphometric method measuring fibrosis quantitatively through software, may fulfill these requirements. DIA can use segmentation and a pixel counting process to measure areas of fibrosis and overall parenchyma from digital histological images, producing a proportionate area by calculating the proportion of tissue occupied by fibrosis [20]. This computerized technology provides quantitative and objective results represented on a continuous arithmetic scale, rather than by a limited number of descriptive staging categories only [20]. A recent study published in *Hepatology*, has strongly addressed the need for more quantitative methods to evaluate fibrosis changes because current evaluation systems unfortunately lack a well-established assessment for important dynamic changes in fibrosis [19].

Although several studies have attempted to identify the usability of DIA in liver fibrosis assessment, the validation of DIA by a correlation with standard clinical fibrosis scales (particularly early stages) have not been yet established. In this study, we aim to assess the ability of DIA to evaluate early stage liver fibrosis changes and identify the relationship between DIA fibrosis quantification and Scheuer clinical staging system in a cohort of Orthotopic Liver Transplant Patients.

## Materials and methods

### Study design and patient criteria

**Fig 1:**
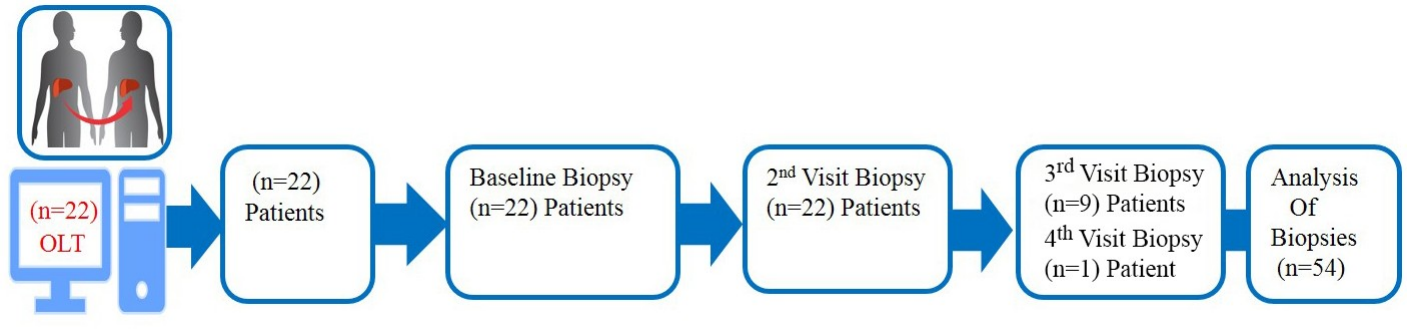
Twenty-two patients had both a baseline biopsy and 2^nd^ biopsy for pathological evaluation; and of those 22, 9 had a 3^rd^ biopsy, and 1 had a 4^th^ biopsy. A total of 54 biopsies were included in the analysis.

A retrospective study at Emory University (Fig 1) was conducted of liver biopsies from 2001 to 2011 from patients who had undergone liver transplantation. The Emory University Hospital digital medical records were accessed April 2012 to July 2020; and biopsies studied were performed June 2004 to July 2011. Inclusion criteria included: patients undergoing liver transplantation for hepatitis C virus infection with at least two post-transplant follow up visits and histopathologic stage of ≤ 2 in the pathology report of their initial liver biopsy using the Scheuer scoring system and no previous or concomitant anti-HCV therapy. Exclusion criteria were as follows: liver comorbidity including hepatitis delta superinfection, hepatitis B virus co-infection, chronic alcohol consumption (< 30 g of pure alcohol per day), Wilson disease, HIV co-infection, or autoimmune hepatitis. The study was approved by the Emory University Institutional Review Board (IRB); and due to the retrospective nature of the study, a waiver of consent was granted by the IRB. Upon analysis, patient data were fully anonymized.

### Sampling procedures and technique

Liver biopsy specimens were fixed in 10% buffered formalin, embedded in paraffin. Histologic sections were cut at 5 μm and stained with Masson’s trichrome and hematoxylin and eosin (H&E). All sections were staged histologically using the criteria of Scheuer et al. [21], ranging from 0 to 4 by an experienced histopathologist. Biopsy specimens were excluded when there was suboptimal assessment due to either inadequate material or documented fragmentation in the final pathological report. Biopsy specimens that meet material adequacy are those that have a core length of at least 10 mm and at least 5 portal tracts.

### Computer-assisted morphometric digital image analysis

**Fig 2:**
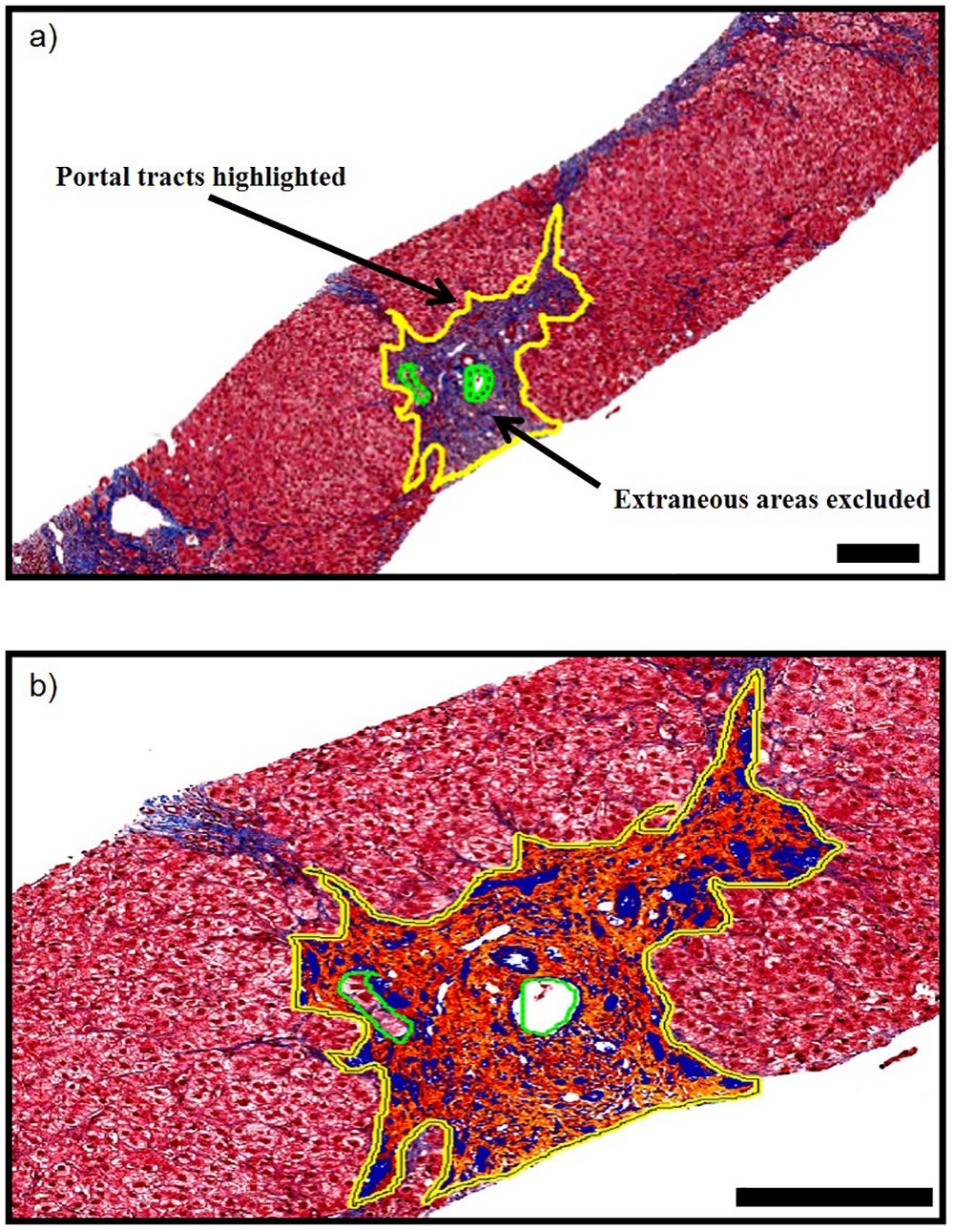
a) A trichrome stain shows how portal tracts were manually delineated (in yellow) and extraneous areas were excluded (in green). The delineated area was the result recorded for the **portal tract area**. b) Tissue considered “positive” is marked up either yellow, orange, or red, in that order with increasing positivity of match to the algorithm parameters. The area measured as “positive” gave the result for the **portal tract fibrotic percentage (%)**. (The scale bar in the lower right corner of each image is 200 μm.).

Trichrome-stained sections were scanned, uploaded into an Aperio ScanScope CS (Aperio Technologies, Inc., Vista, CA), and analyzed using the Leica Aperio Image Scope Positive Pixel Count (PPC) algorithm with scanning magnification and quantitation at 40x (i.e. 20x with 2x magnification doubler) with a numerical aperture of 0.75, giving a 40x resolution of 0.25 μm/pixel. Portal tract area and portal tract fibrotic percentage (%) were quantified by a PPC algorithm with hue measurements tuned to detect fibrosis (Fig 2). Portal tracts were delineated with the Aperio ImageScope pen tool; and the fibrotic portal tract area for each case was obtained in μm. After running the PPC algorithm on the selected portal tract areas, the percentage of fibrosis (fibrosis index) was determined by the ratio of the fibrosis area to the total sample area, expressed in pixels, and calculated automatically from the PPC algorithm output.

### Clinical fibrosis stage

The Scheuer system was used to determine the clinical fibrosis score [21]. Histologic findings of portal inflammation, interface hepatitis, and lobular inflammation are assigned a score ranging from 0 to 4. A separate score from 0 to 4 is assigned to fibrosis stage.

### Statistical analysis

Microsoft Excel (Microsoft Corporation, Redmond, WA) was used to record data and perform preliminary statistical analysis. All data were analyzed with the statistical packages SAS JMP version 12.0.0 (SAS Institute, Cary, NC) and GraphPad Prism 5.0 (GraphPad, La Jolla, CA). Continuous variables were summarized as mean ± standard error [S.E.], and categorical variables as frequency and percentage. Correlation between variables was evaluated by linear regression and Spearman correlation. Statistical significance between groups was determined using Student’s t-test for quantitative measures. A p value < 0.05 was considered significant.

## Results

### Liver biopsies and Scheuer fibrosis score

**Fig 3:**
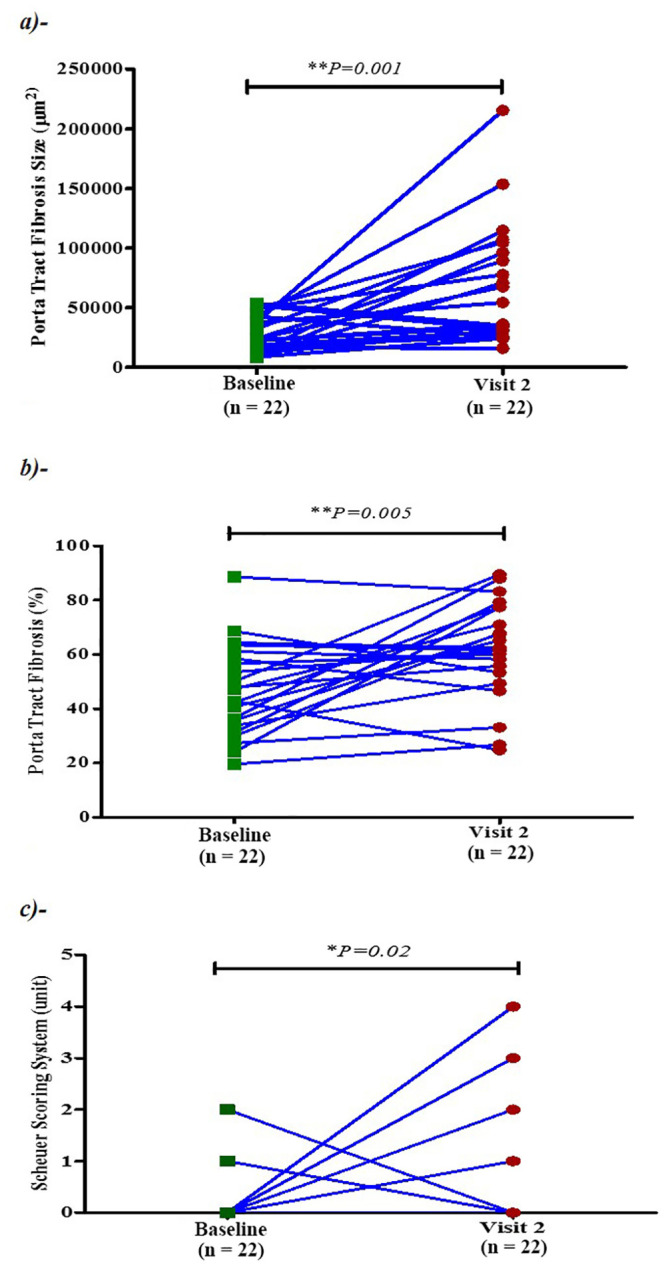
a) Twenty-two patients on average had an increase in the portal tract area (P = 0.001), b) portal tract fibrosis percentage (P = 0.005), and c) Scheuer Scoring System (P = 0.02).

Of the 22 patients who met the inclusion criteria, 54 biopsies were included in the study (Fig 1). Patient and biopsy characteristics are depicted in Table 1. All patients had a liver transplant conducted for hepatitis C virus infection. All 22 had a first (“baseline”) and second follow up biopsy, 9 had a third follow up biopsy, and 1 had a fourth follow up biopsy. All of the liver biopsies showed evidence compatible with recurrent hepatitis C virus infection. The mean biopsy analysis core length was 2.1±1.3 cm (mean ± standard error [S.E.]). Biopsy analysis core length ranged from 0.77 to 4.8 cm. Although all biopsies had a gross length greater than 1.0 cm as part of our inclusion criteria for the study, the digital image analysis length was less than 1.0 cm in 4 biopsies after exclusion of the liver capsule and other extraneous tissues. The patients had their liver transplant at 52.0 ± 1.6 (mean ± standard error [S.E.]) years old and were mostly male (73%). Biopsies were conducted at age of 53.3 ± 1.0 years (mean ± S.E.) and 518 ± 78 days (mean ± S.E.) after transplantation, respectively. At baseline, 70% of liver biopsies (n = 16) were at stage zero of fibrosis, 15% at stage 1, and the same proportion at stage 2. Mean ± S.E. levels of Scheuer fibrosis scores progressed from 0.55±0.19 on the first biopsy (at “baseline”) to 1.14±0.26 at the second visit (*P = 0*.*02)* (Fig 3), while there was no significant difference in fibrosis scores in the second visit when compared to the third or fourth biopsy.

**Table 1:** Patient and biopsy characteristics are depicted below.

*Variable*	Variable Statistic
	(in *n* = 22 patients, 54 biopsies)
***Age***	
*At transplant (Years; AVG ± SE); Range*	52.0 ± 1.6; 31.2 to 66.7
*At biopsy (Years; AVG ± SE); Range*	53.3 ± 1.0; 31.5 to 67.6
***Days to biopsy after transplant (AVG ± SE); Range***	518 ± 78; 30 to 2,616
***Gender (% of Participants)***	
*Female*	27%
*Male*	73%
***Liver Function Measures at time of biopsy (AVG ± SE)***	
*AST (unit/L)*	86.3±17.7
*ALT (unit/L)*	94.4±18.5
*Bilirubin (mg/ dL)*	3.2 ±0.9
*Alkaline phosphatase (unit/L)*	183.8±21.3
*GGT (unit/L)*	160.2±51.8
***Analysis length (cm) (AVG ± SE; Range)***	2.1±1.3; 0.8 to 4.8
***Liver Fibrotic Measures (AVG ± SE; Range) on First Biopsy***	
**Scheuer fibrosis stage**	0.55±0.19; 0 to 4
**Portal tract fibrosis (%)**	46.5 ± 3.6; 19.6 to 88.6
**Portal tract size (**μm^2^)	28,075± 3,232; 8,583 to 53,653
***Liver Fibrotic Measures (AVG ± SE; Range) on Second Biopsy***	
**Scheuer fibrosis stage**	1.14±0.26; 0 to 4
**Portal tract fibrosis (%)**	61.8±3.8; 24.8 to 89.5
**Portal tract size (**μm^2^)	67,146±10,639; 16,002 to 215,486

AST: Aspartate Aminotransferase, ALT: Alanine Aminotransferase, AVG: Average; GGT: Gamma-Glutamyl Transferase, SE: Standard Error.

### Portal tract fibrotic percentage (%) and Scheuer fibrosis score

**Fig 4:**
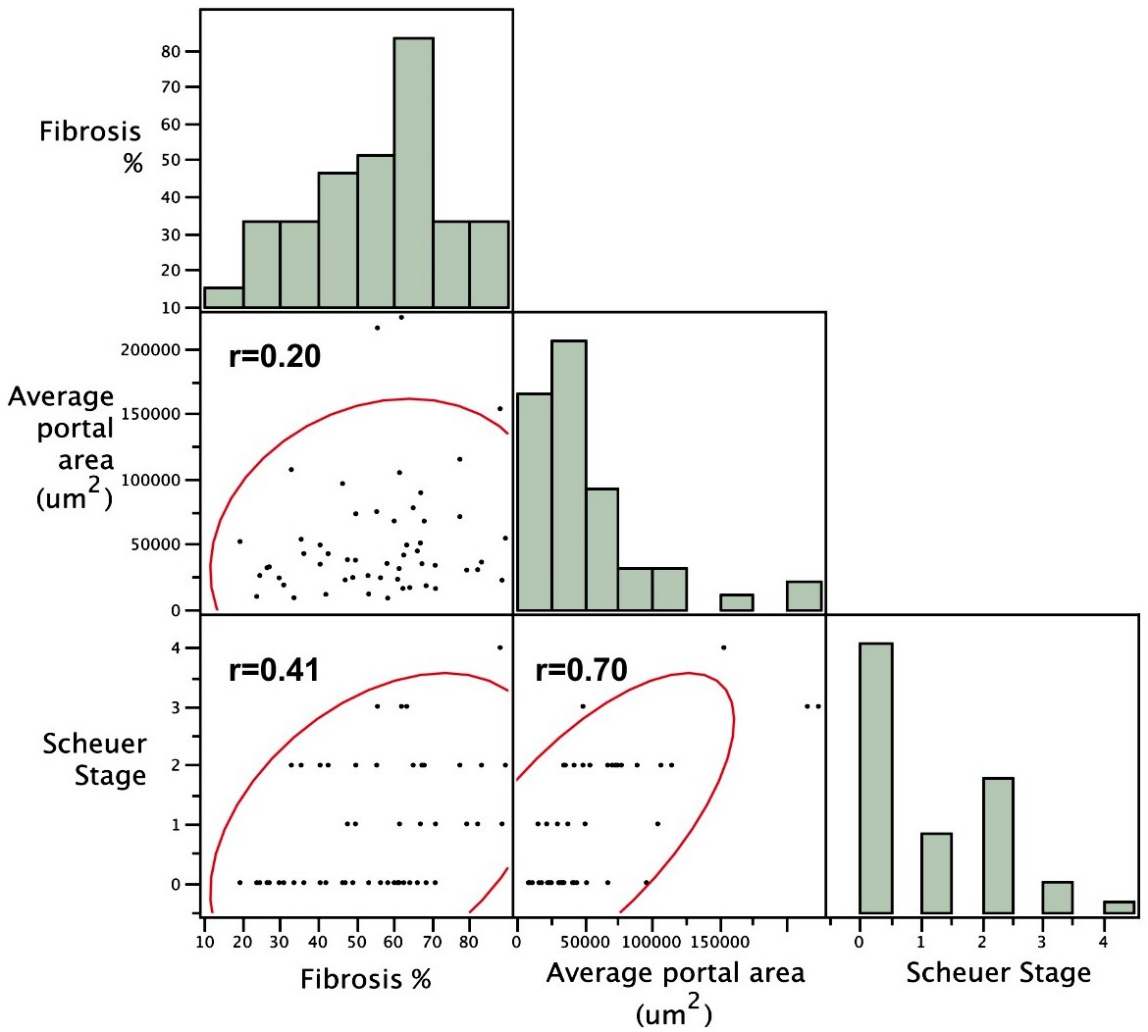
A multivariate analysis with linear regression lines is shown for the specified parameters with the Spearman correlation coefficient r values for each correlation. Histograms depict the measurement distribution for each parameter. The curved lines bound a density ellipse containing 95% of the measurements obtained. Portal tract fibrotic percentage (%) and portal tract area were directly correlated with clinical Scheuer fibrosis stage, with r and P value computed as r = 0.70, P< 0.0001 and r = 0.41, P = 0.002, respectively. The relationship between fibrotic percentage and portal tract area was not statistically significant.

Mean portal tract fibrotic percentage (%) progressed from 46.5 ± 3.6% (mean ± S.E.) at baseline to 61.8 ± 3.8% (mean ± S.E.) at the second visit (*P* = 0.005). Portal tract fibrotic percentage (%) is strongly correlated with clinical Scheuer fibrosis stage, with r = 0.70 (*P* < 0.0001) (Fig 4). Based on the regression analysis, Scheuer fibrosis stage is predicted by portal tract fibrotic percentage with the following equation:

Scheuerfibrosisstage=0.0246×(Portaltractfibrotic%)−0.464

### The portal tract area (μm^2^) and Scheuer fibrosis score

Mean portal tract area progressed from 28,075± 3,232 μm^2^ (mean ± S.E.) at baseline to 67,146 ± 10,639 μm^2^ (mean ± S.E.) at the second visit (P = 0.002). Portal tract fibrotic size (μm^2^) correlated directly with clinical Scheuer fibrosis stage, with r = 0.41 (*P* = 0.002) (Fig 4). Based on the regression analysis, Scheuer fibrosis stage is predicted by portal tract area with the following equation:

$$\text{Scheuer}\;\text{fibrosis}\;\text{stage}=(1.68\times 10^{-5})\times (\text{Portal}\;\text{tract}\;\text{area})+0.0522$$

## Discussion

Conventional histopathological assessment of liver fibrosis is the “gold standard” for allograft monitoring. It is considered a relevant part of patient care and the key to decision making. However, histopathological staging tools are subject to considerable interobserver variability that could lead to uncertainties in judgment regarding the fibrosis degree and management of the fibrosis. Several studies have attempted to improve the diagnostic accuracy using the advanced technologies for whole-slide microscopy images of tissue sections. Although these studies have reported a correlation between the clinical staging score and collagen surface density obtained by image analysis, early fibrosis stages were not a major focus of these studies. [22, 23]. In this study, we validated a DIA technique for fibrosis quantification in liver biopsies of HCV patients that is capable of monitoring their post-transplant fibrotic stage [24].

Current histological staging systems build upon the initial Knodell fibrosis score. These are either 5-tier (Scheuer, Batts-Ludwig, METAVIR, Brunt et al, and Kleiner et al) or 7-tier (Ishak et al); and fibrosis is scored from 0–4 or 0–6, respectively [18, 25]. Staging scores for fibrosis such as the METAVIR, Ishak and Scheuer systems were created to reduce observer variation in liver biopsy evaluation. In the vast majority of clinicopathological studies, liver biopsies with fibrosis score ≥2/4 are considered to be in a critical stage of fibrosis [26]. Thus, cut-off value of ≤ 2 was selected in our study because timely diagnosis of alterations in the normal post-transplant course is a critical factor to minimize morbidity and mortality and to improve outcomes. Generally, grading and staging of liver disease are essentially subjective to interpreter errors, accounting for 15–33% of variability in staging of fibrosis, and 10% of grading of necroinflammation [10, 27].

Digital imaging analysis techniques have been increasingly implemented in histopathological research of liver fibrosis and other histologic studies because of the increased demand for an objective method that does not output ordinal descriptive stage categories merely depending on pathologists’ visual interpretation experience [28]. One important reason for the increased interest in computerized image morphometry in liver pathology is its obvious advantage in quantitative characterization of subtle but clinically relevant fibrosis progression dynamics and prediction of clinical outcomes with different treatments [17]. DIA of the liver samples involves color image representation, image sharpening with noise removal, segmentation, artifact removal, and feature computation (area, number, shape formation, etc.) [20]. Depending on the image quality, image sharpening and noise removal may not be necessary [20]. In our study for liver fibrosis quantification, we used a pixel counting algorithm to quantitatively characterize the intended regions in the digital images.

Our study demonstrates that quantitative measurements from DIA is able to differentiate different stages of liver fibrosis, including early stage fibrosis (0, 1, and 2) with direct correlation to the qualitative assessments of liver fibrosis using the Scheuer staging system. Similar to our study results, Chevalier et al. reported correlation between the clinical Knodell score and the collagen surface density obtained by semi-quantitative morphometric analysis; however, they did not employ computerized analysis, as computerized techniques were not readily available at the time of their study [22]. Moreover, Manousou *et al*, documented a correlation of histological progression of fibrosis using collagen quantification to the rate of increase of clinical fibrosis stage [17]. Interestingly, the DIA quantification method was better in predicting the clinical outcomes of 155 patients with post-transplant liver fibrosis [17]. Like our study, their study employs DIA. However, the investigators employed Sirius red stains; and they did not utilize the fibrotic area percentage method on the more routinely performed trichrome stains utilized in our study. In addition, a recent report by Caballero et al [29] has documented a strong correlation of quantitative morphometric parameters of the portal, periportal and septal fibrosis area with Scheuer fibrosis stages [29]; however, this study evaluated fibrotic stages in native livers and not the transplanted livers, which is the focus of our study. In our study, we have also selected the Scheuer staging system because of its easy applicability and the detailed quantitative information it provides on liver fibrosis to correlate with DIA quantitative data [29].

DIA uses segmentation and a pixel counting process to measure the fibrotic areas and parenchyma from digital histological images, calculating the proportion of fibrosis. Supported by our results, if resources are available, we recommend DIA for liver fibrosis stage evaluation. However, large-scale studies are still needed to evaluate fibrosis before and after transplantation. DIA can serve as an added evaluation and is not a substitute for a descriptive evaluation of architectural changes in the liver. It potentially reduces the assessment variability resulting from pathologists’ subjective visual interpretations [22, 23], and represents a reliable and convenient vehicle for fibrosis evaluation, which is mandatory for clinical management. For example, Sun et al, documented well that quantitative assessment of liver fibrosis reveals precise outcomes in Ishak “stable” patients on anti-HBV therapy [30].

Although technologies for digitizing tissues have advanced significantly during the last decade becoming increasingly feasible for clinical practice, they have not been widely adopted in clinical practice; and this could be attributed to the relatively high cost of the scanning hardware and analysis software or the lack of methodological standardization [30]. To partly address these limitations, we are actively developing whole-slide image analysis algorithms for fibrosis and steatosis quantification that can be accessible by a web platform under development (https://dp.gsu.edu) [31, 32]. In this way, clinicians and researchers with access to the internet can readily invoke our algorithms from a web browser, without the necessity of having image analysis expertise or large-scale computational infrastructure.

Although our study is limited by the relatively small sample for analysis, we were able to use DIA to evaluate early stage fibrosis, which is critical in monitoring and management of liver fibrosis progression/regression. We believe that this current study will serve as an example for groups with similar capabilities, since WSI implementations are likely to increase in the coming years, as we and our collaborations have recently demonstrated [33]. Large-scale quantitative studies are still needed to set a standardization and validation for complete readiness and full applicability of DIA to all clinical stages of liver fibrosis. Efforts are currently underway in our group to extend these methods to artificial intelligence (AI)/machine learning methods that will provide further precision in the interpretation of fibrosis in liver biopsies. For example, other structures in the portal tract could confound analysis (e.g., portal inflammation, bile ducts, or blood vessels); and we are currently developing AI/machine learning methods to improve segmentation and exclusion of extraneous structures. Our group has recently demonstrated the utility of such technology for the analysis of steatosis, utilizing a novel deep-learning approach [34]; and we plan to implement similar techniques for fibrosis analysis in the future. The current study lays the groundwork for our group and other groups to provide improved, precision medicine-type histopathologic analysis of liver biopsies and ultimately enhanced patient care.

## Conclusion

Digital quantitative assessment of portal tract area and fibrosis percentage demonstrates a strong correlation with visually assessed histologic stage of liver fibrosis and complements the standard assessment for allograft fibrosis monitoring. DIA could be considered as a reference for fine fibrosis staging and monitoring sensitive quantitative fibrotic changes of sequential liver biopsies in post-transplant settings because it is both a quantitative and continuous measure of fibrosis. The DIA technique is a reliable method for liver fibrosis quantification that can be applied in clinical practice as a complementary tool to traditional histological methods.

## Abbreviations

DIA: Digital Image Analysis
FDA: Food and Drug Administration
HCV: Hepatitis C Virus
IQR: Interquartile Range
LT: Liver Transplantation
NAFLD: Nonalcoholic Fatty Liver Disease
NASH: Nonalcoholic Steatohepatitis
PPC: Positive Pixel Count
WSI: Whole Slide Image
